# Prognostic and Pathophysiologic Significance of IL-8 (CXCL8) in Biliary Atresia

**DOI:** 10.3390/jcm10122705

**Published:** 2021-06-18

**Authors:** Nimish Godbole, Iiris Nyholm, Maria Hukkinen, Joseph R. Davidson, Athanasios Tyraskis, Katja Eloranta, Noora Andersson, Jouko Lohi, Päivi Heikkilä, Antti Kyrönlahti, Marjut Pihlajoki, Mark Davenport, Markku Heikinheimo, Mikko P. Pakarinen

**Affiliations:** 1 Pediatric Research Center, Children’s Hospital, University of Helsinki and Helsinki University Hospital, 00029 Helsinki, Finland; nimish.godbole@helsinki.fi (N.G.); iiris.nyholm@helsinki.fi (I.N.); maria.hukkinen@hus.fi (M.H.); katja.eloranta@helsinki.fi (K.E.); noora.andersson@helsinki.fi (N.A.); antti.kyronlahti@helsinki.fi (A.K.); marjut.pihlajoki@helsinki.fi (M.P.); markku.heikinheimo@helsinki.fi (M.H.); 2 Section of Pediatric Surgery, Pediatric Liver and Gut Research Group and Pediatric Research Center, Children’s Hospital, University of Helsinki and Helsinki University Hospital, 00029 Helsinki, Finland; 3 Department of Pediatric Surgery, GOS-UCL Institute of Child Health, London WC1N 1EH, UK; joseph.davidson@doctors.org.uk; 4 Department of Pediatric Surgery, King’s College Hospital, London SE5 9RS, UK; thanost88@gmail.com (A.T.); markdav2@ntlworld.com (M.D.); 5 Department of Pathology, University of Helsinki and Helsinki University Hospital, 00029 Helsinki, Finland; jouko.lohi@hus.fi (J.L.); paivi.heikkila@hus.fi (P.H.); 6 Department of Pediatrics, Washington University in St. Louis, St. Louis, MO 63130, USA

**Keywords:** biomarker, cholangiocyte, ductular reaction, liver fibrosis, pediatric liver disease

## Abstract

Interleukin (IL)-8 (CXCL8), a chemokine involved in neutrophil recruitment, has been implicated in ductular reaction and liver fibrogenesis. We studied liver and serum IL-8 expression in a large biliary atresia (BA) cohort and explored its prognostic and pathophysiological potential. IL-8 expression was assessed in liver utilizing quantitative polymerase chain reaction (qPCR), immunohistochemistry and in situ hybridization and in serum using an enzyme-linked immunosorbent assay, among 115 BA patients, 10 disease controls and 68 normal controls. Results were correlated to portoenterostomy (PE) outcomes, biochemical and histological liver injury, transcriptional markers of fibrosis and cholangiocytes, and expression of other related cytokines. IL-8 was markedly overexpressed in liver and serum of BA patients at PE (*n* = 88) and in serum samples obtained during postoperative follow-up (*n* = 40). *IL-8* expression in the liver was predominantly in cholangiocytes within areas of ductular reaction. Liver *IL-8* mRNA expression correlated positively with its serum concentration, bile ductular proliferation, Metavir fibrosis stage, and transcriptional markers of activated myofibroblasts (*ACTA2*) and cholangiocytes (*KRT19*). Taken together, IL-8 may mediate liver injury in BA by promoting ductular reaction and associated liver fibrogenesis. Prognostic value of serum IL-8 to predict native liver survival was limited and confined to the postoperative period after PE.

## 1. Introduction

Biliary atresia (BA), presenting in the neonatal period, is characterized by fibro-inflammatory obliteration of the extra- and intrahepatic bile ducts. Although its exact etiology remains elusive, previous studies have at times suggested a developmental, dysfunctional immune response or environmental toxin hypotheses either individually or in some combination [1,2]. Untreated, the resulting bile duct disruption and cholestasis rapidly progresses to fatal biliary cirrhosis and end-stage liver disease within 2 years from birth [2,3]. The current management of BA involves an early attempt at restoration of bile flow with excision of the obliterated extrahepatic bile ducts and biliary reconstruction using a Roux jejunal loop (portoenterostomy (PE)) [2]. Consequent normalization of serum bilirubin levels is then regarded as a successful outcome. Although PE is successful in over half of the patients, progressive fibrosis continues in their native livers necessitating liver transplantation (LT) in the majority of patients before the age of 20 years [3]. Consequently, BA is the leading cause of LT in children [4]. 

The highly variable surgical prognosis of BA necessitates intensive postoperative monitoring [5]. Reliable tools for prediction of the outcome of PE and progression of liver injury are limited [5,6], but urgently needed for individualized patient follow-up, family counselling and early prediction of the need for LT as well as interventional studies [7]. A chemokine, interleukin (IL)-8 or CXCL8, mediates innate immune activation and regulates neutrophil recruitment and degranulation [8]. Increased IL-8 expression has been linked with reactive cholangiocytes in ductular reaction (DR) and progression of liver fibrosis in other chronic liver diseases in accordance with the ability of IL-8 to induce alpha-smooth muscle actin (α-SMA), a marker of myofibroblast activation [9,10]. Numerous studies have shown that *IL-8* expression is increased in patients with BA and experimental data suggest that IL-8 might contribute to disease progression [8,11,12,13]. However, a recent study has reported that tissue and serum levels of IL-8 of BA patients at the time of PE were not related to the outcome [14]. Thus the pathophysiological significance and the role of IL-8 as a biomarker for liver injury and thereby its predictive ability for PE outcomes has not been yet fully established [3]. 

The aim of this study was to explore the prognostic and pathophysiological potential of IL-8 in BA by assessing its serum and liver expression in a controlled manner. We hypothesized that by correlating with liver fibrogenesis, *IL-8* expression could serve as a valuable prognostic biomarker for the success of PE and native liver survival thereafter. To this end, we assessed liver expression and serum levels of IL-8 and other connected cytokines including IL-18, IL-33, tumor necrosis factor-alpha (TNFα) and interferon gamma (IFN-γ) at PE. We extend previous knowledge by addressing *IL-8* expression also during postoperative follow-up and by relating IL-8 in a large patient cohort to histological liver fibrosis, bile ductular proliferation and transcriptional markers indicative of active liver fibrogenesis and cholangiocytes as well as surgical outcomes.

## 2. Materials and Methods

### 2.1. Patients and Controls

This was a retrospective observational study. We included all BA patients with stored serum or liver samples, who had undergone PE at King’s College Hospital, London, UK, during 2005–2013 or the Children’s Hospital, University of Helsinki, Finland during 2012–2018. Of the 115 included patients representing 33% and 90% of all patients operated on in London and Helsinki during the same eras, 75 had available samples obtained at PE, 27 during post-PE follow-up and 13 at both time points (Table 1). Follow-up samples were collected from stable patients in Helsinki during 2012–2018, where serum samples were obtained at least yearly and liver biopsies at 1 year post-PE and once in 5 years thereafter as a part of routine clinical follow-up [15]. Wedge liver biopsies were taken at PE, and ultrasound guided core needle biopsies under general anesthesia for endoscopic variceal surveillance as described previously [15]. Liver biopsy is not part of the clinical management of patients post-operatively at King’s College Hospital. All PE surgeries were open and postoperative steroids, ursodeoxycholic acid and antibiotics were routinely used in both centers [15]. The diagnosis of BA was confirmed by histopathological assessment of bile duct remnants in both centers. 

Serum samples from 68 generally healthy pediatric day surgery patients and 10 pediatric donor liver biopsies were used as normal controls, and 10 liver biopsies from children with other cholestatic disorders as disease controls. Clinical details of disease controls are displayed in Supplementary Table S1.

### 2.2. Liver Biochemistry and Histology

Serum levels of bilirubin, alanine aminotransferase (ALT), aspartate transaminase (AST), AST-to-platelet ratio index (APRi) and gamma-glutamyl transferase (GGT) were measured by the local hospital laboratories. Liver biopsies were graded for fibrosis using the Metavir staging and scored for cholangiocyte marker cytokeratin (CK)-7 positive bile ductular proliferation (0–2) and portal inflammatory cell infiltration (0–3) by an experienced pediatric liver pathologist blinded to the clinical data as described previously [15,16]. 

### 2.3. Serum Cytokine Levels

Serum concentrations of IL-8, IL-18 and TNF-α were determined using commercially available Q-Plex multiplex ELISA (enzyme-linked immune sorbent assay) array kits (Quansys Bioscience, Logan, UT, USA) following the manufacturer’s instructions.

### 2.4. Liver mRNA Expression 

RNA from liver biopsies was extracted using the RNeasy Mini Kit (QIAGEN, Frederick, MD, USA). RNA concentration was assessed spectrophotometrically. mRNA expression of IL-8, IL-18, IL-33, TNF-α, IFNG, COL1A2, ACTA2, KRT7 and KRT19 were analyzed in triplicate by quantitative real-time polymerase chain reaction (qPCR) using Custom RT Profiler PCR Array (CAPH12366A) (QIAGEN SABiosciences, Frederick, MD, USA) on BIO-RAD CFX384 Real-Time System (Bio-Rad, Hercules, CA, USA) according to the manufacturer’s instructions. ACTA2 (marker of myofibroblast activation) and COL1A2 (marker of collagen production) were studied as surrogates for active liver fibrogenesis, and KRT7 and KRT19 as cholangiocyte markers, while HPRT1, GAPDH, ACTB and B2M were used as housekeeping genes. Quantification of target gene mRNA expression was performed using the ΔΔCt method and expressed after normalization to housekeeping genes and relative to normal control subjects.

### 2.5. Immunohistochemistry

Formalin fixed, paraffin embedded (FFPE) sections were deparaffinized, hydrated, and treated with target retrieval solution pH 9 (Dako - Agilent Technologies, Glostrup, Denmark). Commercially available antibody for IL-8 (rabbit polyclonal, AHC0881, ThermoFisher Scientific, Waltham, MA, USA) was used at a dilution of 1:3000 along with the Novolink TM polymer detection system (Leica biosystems Newcastle Ltd, Newcastle Upon Tyne, UK). Primary antibody was incubated overnight at 40 °C. Images were generated using 3DHISTECH Panoramic 250 FLASH II digital slide scanner at Genome biology unit supported by HiLIFE and the Faculty of Medicine, University of Helsinki, and Biocenter Finland.

### 2.6. RNA In Situ Hybridization 

RNA in situ hybridization was performed on fresh 4.5 μm FFPE tissue sections using RNAscope Multiplex Fluorescent Reagent Kit Version 2 (#323100, Advanced Cell Diagnostics, Newark, CA, USA) for target detection according to the manual. Tissue sections were baked for 1 h at 60 °C, then deparaffinized and treated with hydrogen peroxide for 10 min at room temperature (RT). Target retrieval was performed for 15 min at 98 °C, followed by protease plus treatment for 15 min at 40 °C. All probes (Hs-ONECUT1 (HNF6) #490081, Hs-IL-8 #310381, 3-Plex negative control probe dapB #320871 and 3-plex positive control probe, POLR2A, PPIB, UBC #320861, Advanced Cell Diagnostics, Newark, CA, USA) were hybridized for 2 h at 40 °C followed by signal amplification and developing of HRP channels undertaken according to the manual. TSA Plus fluorophores fluorescein (1:1000 dilution), Cyanine 3 (1:1500 dilution) and Cyanine 5 (1:3000 dilution) (NEL744001KT, PerkinElmer, Waltham, MA, USA) were used for signal detection. The sections were counterstained with DAPI (4’, 6-diamidino-2-phenylindole) and mounted with ProLong Gold Antifade Mountant (P36930, Invitrogen ThermoFisher Scientific, Waltham, MA, USA). Tissue sections were scanned using 3DHISTECH Panoramic 250 FLASH II digital slide scanner at Genome Biology Unit (Research Programs Unit, Faculty of Medicine, University of Helsinki, Biocenter, Finland) using 1 × 20 magnification with extended focus and 7 focus levels. 

### 2.7. Statistical Methods

Unless otherwise stated, continuous variables were expressed as medians with interquartile ranges and compared using the Mann–Whitney U test. Multiple group comparisons were undertaken using the Kruskal–Wallis test. Correlations were tested with Spearman’s rank correlation between different variables analyzed from the same liver biopsy or from simultaneously obtained liver and serum samples. To address effects of different expression levels on native liver survival, Kaplan–Meier analysis with log-rank test was used to predict native liver survival between tertiles for serum IL-8 concentration and relative liver mRNA expression. *p* < 0.05 was considered as statistically significant and all analyses were done on RStudio version 1.2.5033 (RStudio, Boston, MA, USA). Numbers throughout the analysis may be discrepant, owing to differing samples available from relevant time points (Table 1). These are given in each section of the Results.

### 2.8. Ethics

The study protocol was approved by the local hospital ethical committees. All procedures followed were in accordance with the ethical standards of the responsibility committee on human experimentation (institutional/national) and with the Helsinki Declaration of 1975, as revised in 2008. The study was approved by the hospital ethical committee (protocol number 345/03/1372008) and the institutional review board on 21 July 2017 (§68 HUS/149/2017) and also by the National Research Ethics Committee of the UK (12/WA/0282 and 18/SC/0058). An informed consent for use of samples in research was obtained from all patients.

## 3. Results

### 3.1. Patient Characteristics

Patient characteristics are listed in Table 1. The median patient age at the time of surgery was 56 days (41–76), and 96% of the children presented with type 3 BA. Following PE, 74 (64%) of the patients normalized their serum bilirubin (<20 µmol/L), and 56 (49%) underwent LT at median age of 1.5 years (interquartile range (IQR) 0.8–3.0). Native liver survival was 77% (95% confidence interval (CI) 70–85), 69% (95% CI 61–78) and 56% (95% CI 47–66) at 1, 2 and 5 years respectively.

### 3.2. Liver Expression and Serum Levels of Interleukin-8 (IL-8) Were Increased and Intercorrelated

**Figure 1:**
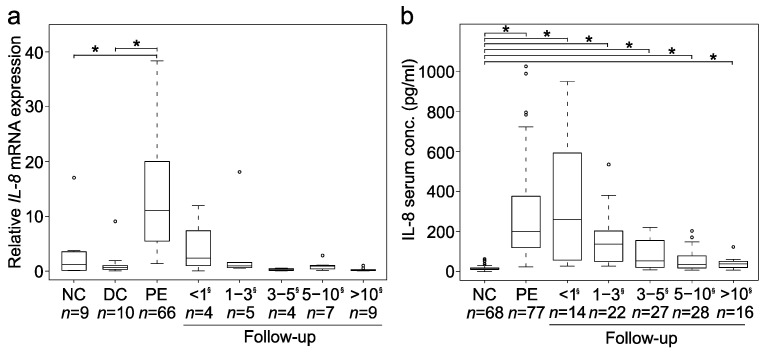
Liver and serum expression of interleukin-8 (IL-8). Box plots (median, interquartile range and 90th percentile) of (**a**) liver IL-8 mRNA expression (fold-change) and (**b**) serum IL-8 concentration (pg/mL) in normal controls (NC), disease controls (DC), biliary atresia patients at portoenterostomy (PE) and during follow-up following PE * *p* < 0.05 ^§^ = Years after PE. circles = outliers.

Liver *IL-8* mRNA expression was markedly increased at PE (*n* = 66), when compared to both disease (by 16-fold) and normal (by 9-fold) control groups (*p* < 0.001) (Figure 1a). Similar to the upregulated liver expression, we found a significant, over 15-fold increase (*p* < 0.001) in IL-8 serum levels at PE (*n* = 77) compared to normal controls (Figure 1b). Both liver mRNA expression and serum levels of IL-8 peaked at and within one year after PE and declined thereafter, although serum IL-8 levels remained significantly above normal control values. A positive intercorrelation (r = 0.53, *p* < 0.01, *n* = 70) was observed between liver mRNA expression and serum concentrations of IL-8 (Supplementary Figure S1).

The patient age at PE positively correlated with liver *IL-8* mRNA expression (r = 0.32, *p* < 0.01, *n* = 66) and IL-8 serum levels (r = 0.31, *p* < 0.01, *n* = 77). At PE, the patients with splenic malformation showed lower liver IL-8 mRNA expression (5.51 (3.95–7.22) fold, *n* = 10) than those without (13.31 (6.37–21.07) fold, *n* = 54, *p* = 0.02), but median serum IL-8 concentrations were similar in patients with (164 (116–225) pg/mL, *n* = 9) and without (208 (121–381) pg/mL, *n* = 68, *p* = 0.44) splenic malformation. *IL-8* liver mRNA expression (14.9 ± 10.7 vs. 11.6 ± 9.92) fold, *p* = 0.288) and serum levels (394 ± 613 vs. 372 ± 204 pg/mL, *p* = 0.823) were comparable in the London and Helsinki samples.

### 3.3. Liver IL-8 Expression Predominantly Localized to Cholangiocytes

**Figure 2:**
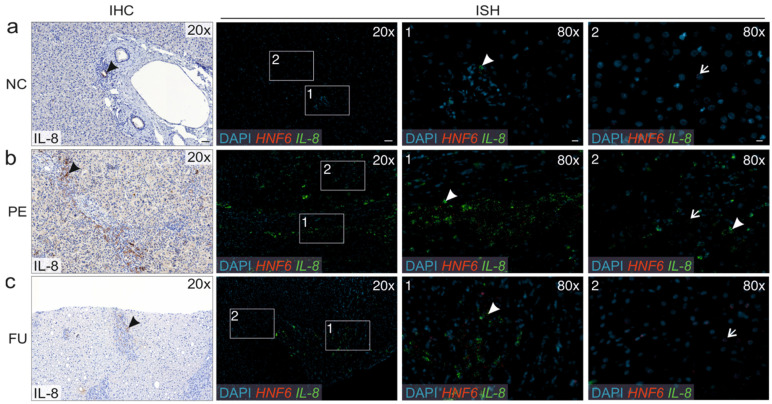
Representative liver expression of *IL-8* on immunohistochemistry (IHC) and in situ hybridization (ISH) in (**a**) normal control (NC), (**b**) biliary atresia patient at portoenterostomy (PE) and (**c**) during follow-up (FU) after PE. ISH includes two magnified images (80×) from each of the original (20×) image focused on (1) the area of ductular reaction (DR) and (2) hepatocyte rich parenchymal area. Arrow heads (black and white filled) point to IL-8 expressing cells (brown in IHC and green in ISH) while arrows (white filled) point to a hepatocyte marker HNF6 expressing cells in ISH. Note the expression of *IL-8* in bile duct cholangiocytes in the normal liver, while IL-8 is strongly expressed in the DR area and cholangiocytes instead of hepatocytes in biliary atresia patients. Scale bar = 50 μm (20×)/10 μm (80×).

As studied with immunohistochemistry *IL-8* expression localized to cholangiocytes in the bile ducts within the normal liver (Figure 2a). In BA livers, enhanced *IL-8* expression was mainly observed in cholangiocytes within areas of DR and also in the cytoplasm of surrounding cells, most likely representing inflammatory cells. *IL-8* expression was more prominent at PE (Figure 2b) than during the follow up (Figure 2c). The findings were confirmed using in situ hybridization for IL8 and a hepatocyte marker HNF6, showing *IL-8* expression in bile duct cholangiocytes in the normal liver, and a predominant *IL-8* expression in the DR areas and cholangiocytes instead of hepatocytes in biliary atresia patients.

### 3.4. IL-8 Associated with Liver Fibrosis, Ductular Reaction and Liver Injury

**Figure 3:**
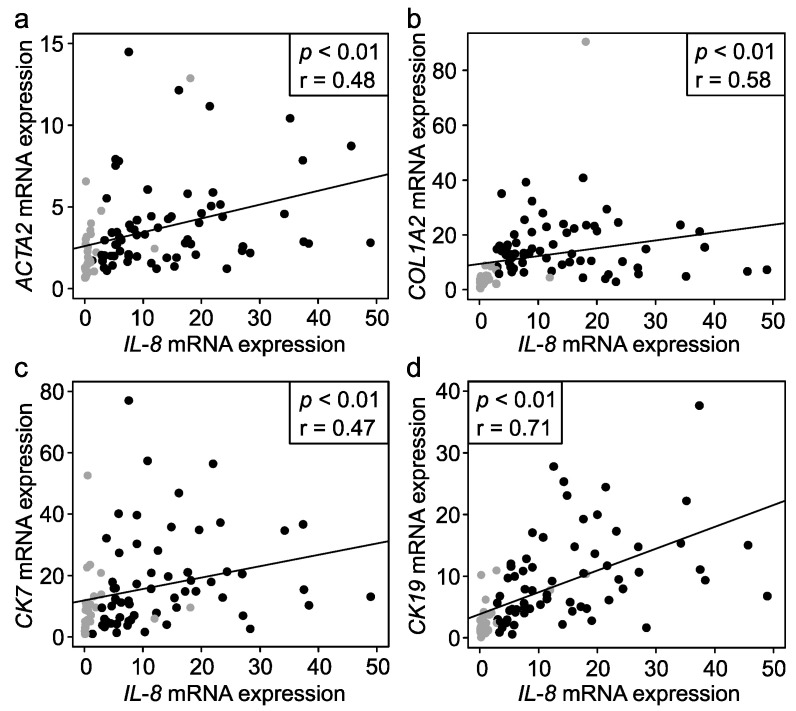
Correlations between liver *IL-8* expression and transcriptional markers of fibrogenesis and cholangiocytes. Scatterplot for correlation between relative liver *IL-8* mRNA expression and (**a**) *ACTA2* (**b**) *COL1A2* (**c**) *KRT7* and (**d**) *KRT19*. Black dots represent samples obtained at portoenterostomy (PE) and grey dots represent follow up samples.

Liver *IL-8* mRNA expression correlated positively with the Metavir fibrosis stage at PE (r = 0.28, *p* = 0.04, *n* = 51) and in follow-up biopsies (r = 0.44, *p* = 0.05; *n* = 19), and with fibrosis markers *ACTA2* encoding for α-SMA and *COL1A2* encoding for collagen type 1 (Figure 3). Liver *IL-8* expression correlated positively with bile ductular proliferation (r = 0.45, *p* < 00.1, *n* = 70) and cholangiocyte markers *KRT19* encoding for CK-19 and *KRT7* encoding for CK-7 (Figure 3). No correlation with portal inflammation score was observed (r = 0.14, *p* = 0.26, *n* = 67). *IL-8* mRNA expression correlated positively with bilirubin (r = 0.51, *p* < 0.01), AST (r = 0.48, *p* < 0.01), ALT (r = 0.53, *p* < 0.01) and GGT (r = 0.51, *p* < 0.0, *n* = 70 for all) and with APRi during follow-up (r = 0.54, *p* = 0.04, *n* = 15). 

At PE, serum IL-8 levels correlated with GGT (r = 0.27, *p* = 0.02, *n* = 77), whereas in the follow up serum samples, IL-8 not only correlated positively with GGT (r = 0.75, *p* < 0.01), but also with bilirubin (r = 0.58, *p* < 0.01), AST (r = 0.71, *p* < 0.01), and ALT (r = 0.69, *p* < 0.01; *n* = 36 for all). Although serum IL-8 levels correlated positively with bile ductular proliferation (r = 0.51, *p* < 0.01, *n* = 101), *COL1A2* (r = 0.29, *p* = 0.01, *n* = 69) and *KRT19* (r = 0.31, *p* < 0.01, *n* = 68), no significant correlations with the Metavir fibrosis stage, portal inflammation score, *ACTA2* or *KRT7* expression were observed. 

### 3.5. IL-8 Had Limited Ability to Predict Portoenterostomy (PE) Outcomes

**Figure 4:**
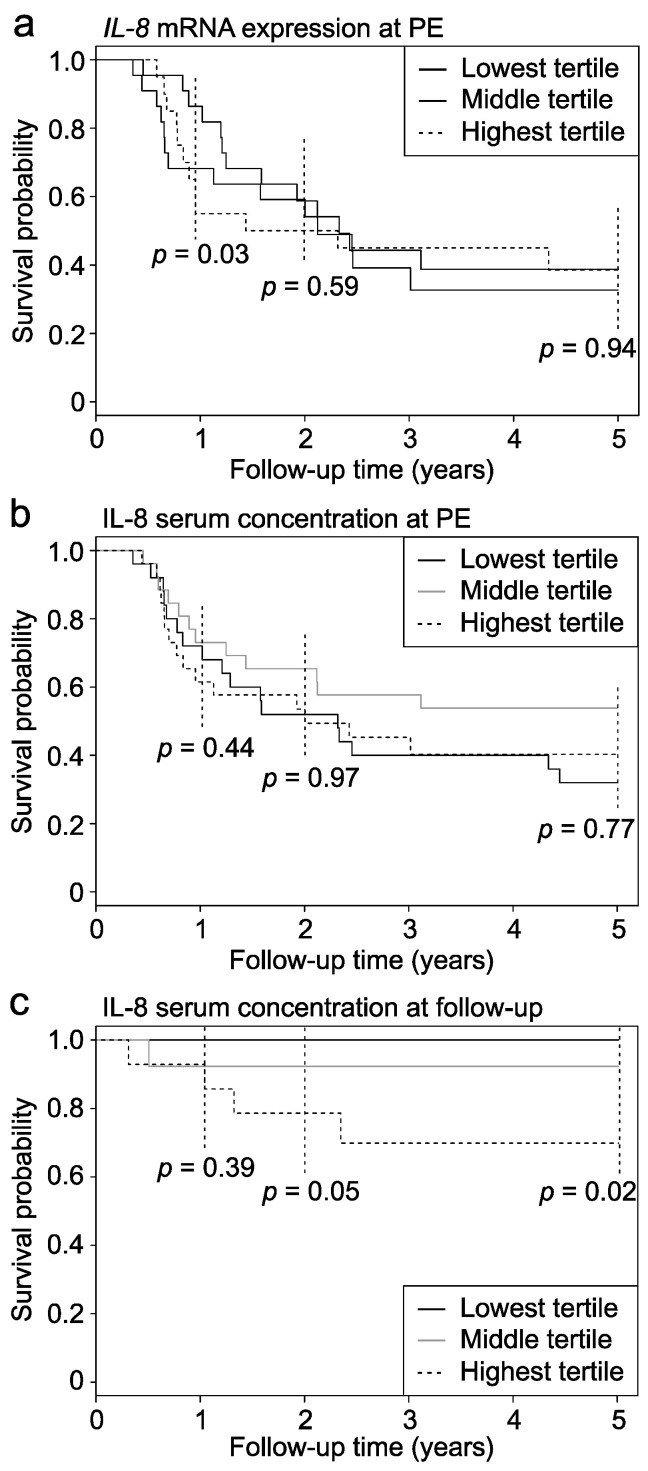
Native liver survival according to tertiles for IL-8 expression. Kaplan–Meier survival curves for native liver survival according to tertiles of (**a**) liver *IL-8* mRNA expression at portoenterostomy (PE) (*n* = 64), (**b**) serum IL-8 concentration at PE (*n* = 77), and (**c**) serum IL-8 concentration measured at the first follow-up sample (*n* = 40).

At PE, median serum levels of IL-8 were similar in patients (*n* = 41) who normalized serum bilirubin after PE (191 (128–327) pg/mL) when compared to those (*n* = 36) who remained jaundiced [208 (112–409) pg/mL, *p* = 0.54]. Liver IL-8 mRNA expression was also not significantly different between patients who cleared their jaundice and who did not (Supplementary Figure S2). However, when divided into tertiles based on their liver IL-8 expression level, the patients with the lowest IL-8 expression at PE showed significantly higher 1-year native liver survival (*p* = 0.03) compared to those with the highest expression, while no difference was seen in their 2- and 5-year native liver survival (Figure 4a). There was no difference in native liver survival rates at 1, 2 or 5 years between serum IL-8 tertiles measured at PE (Figure 4b).

### 3.6. Serum IL-8 Associated with Native Liver Survival during Post-PE Follow-Up

Forty patients underwent median two (range, 1–6) follow-up serum IL-8 measurements after PE. The first follow-up measurement was performed 2.9 (1.1–4.2) years postoperatively, and the median period between the first and last measurement was 3.4 (0.18–5.4) years. Ten of these 40 patients underwent LT 3.8 (1.1–6.2) years after PE, while 30 patients had survived with their native livers for 6.7 (2.9–9.2) years. The first (154 (130–310) vs. 48.0 (32–197) pg/mL, *p* = 0.03), the last (117 (65–269) vs. 35.4 (20–86) pg/mL, *p* = 0.01) and the average (147 (108–289) vs. 44.0 (28–164) pg/mL, *p* = 0.02) postoperative serum IL-8 level was significantly higher among the patients who were later transplanted when compared to those who continued to survive with their native liver. In addition, the patients in the lowest serum IL-8 tertile of the first postoperative measurement showed significantly higher native liver survival than the patients in the highest tertile (Figure 4c).

### 3.7. Expression of Cytokines Connected to IL-8

**Figure 5:**
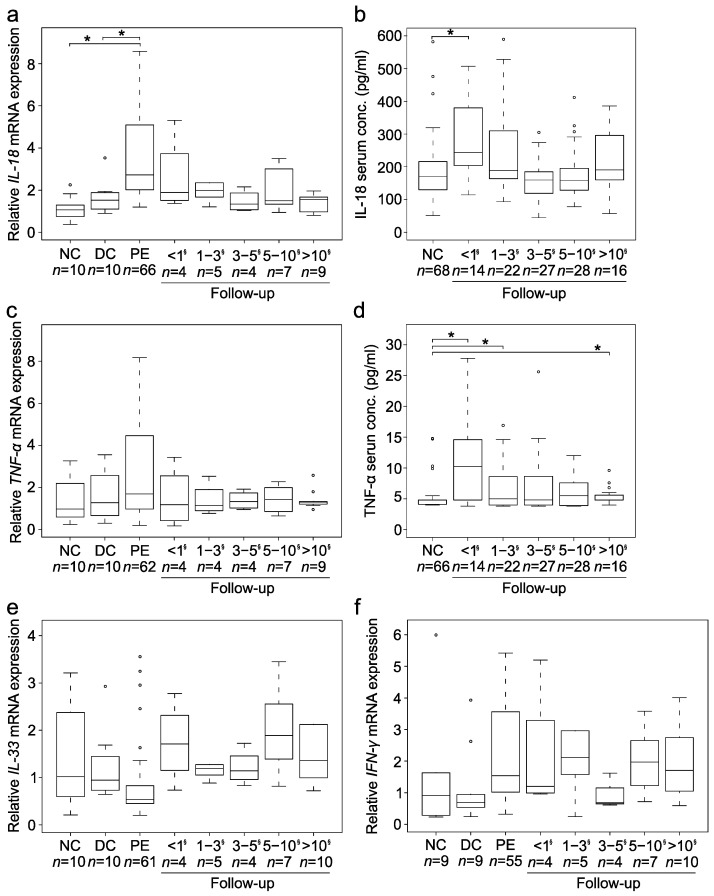
Expression of several cytokines related to IL-8. Box plots (median, interquartile range and 90th percentile) of (**a**) liver *IL-18* mRNA expression (fold-change) and (**b**) serum IL-18 concentration (pg/mL), (**c**) liver *TNF* mRNA expression, (**d**) serum tumor necrosis factor-alpha (TNF-α) concentration (pg/mL), (**e**) liver *IL-33* mRNA expression, and (**f**) liver *IFN* mRNA expression in normal controls (NC), disease controls (DC), biliary atresia patients at portoenterostomy (PE) and during follow-up following PE * *p* < 0.05. ^§^ = Years after PE circles = outliers.

As shown in Figure 5, expression of other cytokines connected to IL-8 changed less consistently, although *IL-18* (*n* = 66) mRNA expression was also significantly increased both at PE (*p* < 0.01) and <1 year after PE (*p* < 0.01). Despite IL-8 correlating positively with IL-18 at transcriptional level (r = 0.56, *p* < 0.01; *n* = 95) and in serum (r = 0.28, *p* < 0.01; *n* = 107), the postoperative *IL-18* expression remained unchanged. While TNF-α showed slightly increased postoperative serum concentration among patients, it along with *IFN-γ* and *IL-33* did not show any significant overexpression at transcriptional level. However, also *TNF-α* (r = 0.40, *p* < 0.01, *n* = 90), *IFN-γ* (r = 0.26, *p* = 0.01, *n* = 84) and *IL-33* (r = 0.46, *p* < 0.01, *n* = 90) positively correlated with *IL-8* mRNA expression in the liver while *TNF-α* also correlated with IL-8 in serum (r = 0.14, *p* = 0.04, *n* = 107).

## 4. Discussion

In this study we have comprehensively addressed the prognostic value of liver and serum expression of IL-8 for PE outcomes in a large number of BA patients in relation to histological liver fibrosis, (transcriptional) markers of liver fibrogenesis and cholangiocytes as well as biochemical markers of liver injury. We demonstrated marked parallel increases in liver and serum expression of IL-8, which were specific for IL-8 among several other cytokines studied. Although liver *IL-8* expression was associated with histological liver fibrosis, and surrogate markers of liver fibrogenesis as well as bile ductular proliferation and cholangiocytes at the mRNA level, serum IL-8 levels were associated with further survival with native liver only in samples obtained during postoperative follow-up after PE. Our findings suggest that although IL-8 may have a distinctive pathophysiological role in BA by promoting DR and liver fibrogenesis, prognostic value of serum IL-8 in predicting PE outcomes is limited to the postoperative follow-up period after PE.

Prolonged immune activation with the release of several proinflammatory cytokines in the affected liver is considered vital for the developing fibrosis and liver damage in BA [17]. At the time of PE, an inflammatory infiltrate associated with DR and fibrosis along the portal tracts occurs [16,18]. Liver fibrosis progresses, also following successful PE surgery, for largely unknown reasons [15], but has been attributed to activation of hepatic stellate cells and portal myofibroblasts by cytokines derived from Kupffer cells and reactive cholangiocytes of DR [3,5,19,20], leading to the deposition of an extra cellular matrix and collagen. In accordance with previous experimental observations [11], our findings suggest that IL-8 is involved with the above outlined liver injury cascade in BA, and that the involvement was specific to IL-8 as several other cytokines studied failed to show enhanced expression to a comparable degree. Not only was *IL-8* distinctively overexpressed in BA livers, but also correlated with histological fibrosis, bile ductular proliferation and transcriptional markers of fibrogenesis and cholangiocytes. In addition, liver *IL-8* expression was localized to cholangiocytes in the areas of DR in accordance with previous findings in adult cholestatic liver diseases [21]. We can only speculate as to the underlying reasons for the lower correlation of liver *IL-8* mRNA expression with the Metavir stage than with ACTA2 or COL1A2. One possible reason could be that the Metavir stage is a class variable as opposed to mRNA expression being a continuous variable, which may skew the correlation between them. There is also a notable overlap between histological scoring of adjoining Metavir stages, especially between stages 2 and 3. Histological portal inflammation score gives a crude estimation of all inflammatory cells present in portal areas, and the missing correlation between portal inflammation and *IL-8* expression might be related to similar limitations as outlined above for histological fibrosis score. 

Overexpression of IL-8 peaked at and within one year after PE and gradually decreased thereafter. The early expression peak may be attributable to the combined effects of overt cholestasis, vigorous bile ductular proliferation and inflammation before surgical re-establishment of bile flow as well as plausibly causative viral infection directly inflicting the initial bile duct damage [1,3,17]. The fact that liver *IL-8* expression showed only limited prognostic value for PE outcomes suggest a non-decisive but complementary role of IL-8 as an indicator for the success of PE or as a mediator for the progression of postoperative liver injury in BA. Contrary to liver *IL-8* expression, serum IL-8 showed no association with histological liver fibrosis and only weak correlations with surrogate markers of liver injury measured by qPCR, explaining the poor predictive value of serum IL-8 at PE. Our findings are in accordance with a recent study among 57 BA patients, where serum and liver IL-8 concentration measured at PE was unrelated to 2-year native liver survival following PE [14].

Interestingly, the increased post-PE expression profile of serum IL-8 followed that of cholangitis episodes, frequency of which is reported to predominate the first postoperative year with a prompt subsequent decline [22,23,24]. It has been previously shown that expression of *IL-8* in human biliary epithelial cells is stimulated by proinflammatory cytokines IL-1β and TNF-α, and lipopolysaccharide produced by gram negative bacteria, which are commonly causative microbes underlying BA associated cholangitis [21,23,25,26]. In the early disease phase, enhanced secretion by an augmented peripheral immature B-cell population seems to be an important source of elevated serum IL-8 [27]. Serum TNF-α and IL-18 levels have been shown to be significantly increased within 6 months of PE surgery [28]. Here, liver and serum expression patterns of TNF-α followed those of IL-8 and their liver mRNA expression correlated with each other. Based on these data, we hypothesize that besides persisting DR, bacterial cholangitis may have contributed to the increased postoperative serum IL-8 levels, and may help to explain their association with native liver survival as recurrent postoperative cholangitis episodes are known to relate with the need of liver transplantation following PE [29]. 

In accordance with previous reports, serum IL-8 levels positively correlated with biochemical liver injury markers such as bilirubin, GGT and transaminases during postoperative follow-up, reflecting progression of cholestasis and liver injury [30]. The significant correlation of IL-8 with bile ductular proliferation and GGT both at PE and during follow-up reinforces the connection between IL-8 and bile duct injury at both time points. We hypothesize that the increased serum IL-8 levels mainly reflected active DR, while postoperative serum levels may have been contributed by bacterial cholangitis. However, the shift from a predominantly inflammatory liver injury phenotype at PE to the one predominated by DR and fibrosis may have also allowed their more specific detection and accurate prediction by serum IL-8 during follow-up [15,31]. 

The main limitations of this study include the retrospective design, which is unavoidably associated with inaccuracies in data collection and missing data points. Although we were not able to prospectively include consecutive patients, PE age, occurrence of associated malformations, anatomic type of BA, clearance of jaundice rate and native liver survival among the included patients were well represented in previously described BA cohorts from Europe [32,33]. While we were able to include a relatively large number of BA patients at PE and during follow-up, the number of patients with simultaneous postoperative liver and serum specimens remained limited. Moreover, serum samples were not collected during cholangitis episodes, precluding our ability to address the actual relationship between serum IL-8 concentration and cholangitis, which should be addressed in future studies. Most of the follow-up serum IL-8 measurements were performed after 2 years of PE, which introduces a selection bias and precludes any conclusions regarding the early postoperative period. Finally, our findings concerning follow-up studies may not be generalizable to patient cohorts with different postoperative treatment regimens.

## 5. Conclusions

Our data showed significant and distinctive overexpression of IL-8 by cholangiocytes within DR in BA patients both at PE and during postoperative follow-up. Despite the IL-8 overexpression in the liver, increased serum IL-8 levels associated weakly with decreased native liver survival only during the postoperative period following PE.

## Figures and Tables

**Table 1:** Baseline patient characteristics and included study samples for all patients (*n* = 115), this included patients with samples only at portoenterostomy (PE) (*n* = 75) and patients with samples only at follow-up (*n* = 27) as well as 13 patients with samples both at PE and follow-up. Data are median (interquartile range) or frequencies (%).

	All Patients (*n* = 115)	Patients at PE (*n* = 88)	Patients at Follow Up (*n* = 40)
Age at PE, days	56 (41–76)	55 (41–75)	56 (35–76)
Follow up after PE, years	3.6 (1.1–9.7)	2.1 (0.7–6.6)	9 (3.6–11.7)
Type of BA, *n* (%)			
1 or 2	5 (4%)	1 (1%)	5 (13%)
3	110 (96%)	87 (99%)	35 (87%)
Splenic malformation, *n* (%)	17 (15%)	11 (13%)	8 (20%)
Cystic disease, *n* (%)	15 (13%)	12 (14%)	5 (13%)
Clearance of Jaundice, *n* (%)	74 (64%)	47 (53%)	38 (95%)
Liver transplantation, *n* (%)	56 (49%)	49 (56%)	10 (25%)
Age at liver transplantation, year	1.5 (0.8–3.0)	1.2 (0.7–2.3)	6.9 (2.5–9.4)
Died without transplantation, *n*	3	3	0
Liver biochemistry			
Bilirubin total, μmol/L	132 (18–167)	145 (124–177)	10 (5–17)
GGT (U/L)	329 (107–673)	572 (235–873)	62 (25–162)
AST (U/L)	165 (94–232)	196 (143–260)	74 (52–122)
ALT (U/L)	84 (44–123)	112 (64–163)	48 (24–98)
APRi	0.84 (0.49–1.43)	0.83 (0.5–1.23)	1.2 (0.48–1.92)
Included serum and liver samples			
Number of patients with Serum IL-8 samples	*n* = 109	*n* = 77	*n* = 40
Number of Serum IL-8 samples/patient	1	1	3 (2–4)
Number of patients with liver biopsies	*n* = 82	*n* = 66	*n* = 22
Number of liver biopsies/patient	1	1	2 (1–2)

BA: biliary atresia; PE: portoenterostomy; GGT: gamma-glutamyl transferase; AST: aspartate transaminase; ALT: alanine aminotransferase; APRi: AST-to-platelet ratio index; IL-8: Interleukin-8.

## Supplementary Materials

The following are available online at https://www.mdpi.com/article/10.3390/jcm10122705/s1, Figure S1: Correlation between liver and serum expression of IL-8, Figure S2: Liver and serum expression of IL-8 in relation to clearance of jaundice after portoenterostomy, Table S1: Baseline characteristics of disease control patients.
