# Supplementary material for: Prognostic and Pathophysiologic Significance of IL-8 (CXCL8) in Biliary Atresia
Source: J Clin Med. 2021 Jun 18;10(12):2705. doi: 10.3390/jcm10122705 (PMC8234515; doi:10.3390/jcm10122705)
Supplement: Supplementary file 1 [file jcm-10-02705-s001.zip › Supplementary/Supplementary Table 1.pdf]

Supplementary Table S1: Baseline characteristics of disease control patients

	Disease controls
Patients, n	10
Female, n (%)	2 (20%)
Age at liver biopsy, days	62 (38 – 1187)
Liver biochemistry	
Bilirubin, µmol/l	102 (38 – 113)
Conjugated bilirubin, µmol/l	55 (33 – 93)
AST, U/l	144 (115 – 161)
ALT, U/l	91 (52 – 131)
APRi	0.6 (0.4 – 0.9)
